# Nursing in East London

**Published:** 1888-03-10

**Authors:** 


### Nursing in East London.

Though the East End generally—and Stepney in particular—has become of late "a garden of romance," the happy hunting-ground of story-tellers and ballad-writers, it can hardly be called picturesque. Around the great thoroughfares of Mile End Road and Commercial Road spreads a network of narrow streets, new and old, some respectable, some the reverse, but all poor and sordid.

It is in this quarter of the world of London, where ignorance, neglect, and insanitary conditions do their bitter worst, that the East London Nursing Society has taken up its abode. Founded in 1868 for the purpose of nursing the sick poor in their own homes, it is gradually extending its area, and every year sees some addition to its scope. It began with placing a nurse in six parishes; now it has twenty-two nurses working under the supervision of three matrons. The society retains the division of the ground it works on into parishes, because this is more convenient, and because thereby it can get help that is essential to the success of the nurses' work. If the incumbent of any parish asks the society to have a nurse placed there, he must guarantee that she shall have a respectable lodging, and that there will be some local fund to supply what comforts in the way of food and stimulants she may require for her patients. In nursing the poor—whose illnesses are often brought on, and oftener still aggravated, by hunger—it is absolutely necessary to supplement the skill of doctor and nurse by some such provision, which, nevertheless, it is not within the province of the society to grant. As the East End is not rich in people who have anything to spare from their own wants, it is from other parts of London that such help must be sought. The cost of establishing a nurse is £60 for the first year, and about £54 per annum afterwards. The matrons have each the supervision of six or seven parishes. Their duty is to see every new case immediately, and to visit both with and without the nurse, thus making sure that these do their work efficiently. The nurse is expected to apprise the matron of her division of each new case she is called to attend, and to keep a register of her work. Each nurse must spend eight hours a day in looking after her patients—the first four hours to be from nine a.m. till one, while the others may be later in the day, to make the patients comfortable for the night.

These being the general conditions of the society's work, let me give a short account of a few cases I visited in company with Miss Taylor, matron of the northern division, whose centre is at the society's office, 49, Philpot Street, Commercial Road.

The weather was just recovering from a severe attack of east wind; there was sunshine enough to make even Stepney look less ugly than its wont, but the air was still cold enough to make one expect to meet with pneumonia, pleurisy, and all the other troubles that cold is apt to develop, especially in ill-clad and ill-nourished frames. The first house we visited held one of these cases—a boy of twelve, whose flushed face showed that the inflammation of the lungs from which he had suffered was not yet wholly conquered. The matron's first demand was for the paper on which it is the nurse's duty to note the patient's temperature—a record which showed that the child had been in great danger, but was now, however slowly, moving towards recovery. The room was poorly furnished, and the mother and a girl, whose *coiffure* was rich in curl-papers, were not notably tidy in their appearance; but a bright fire burned in the grate—thanks to a gift of coals which had been received from the church—and the patient himself was perfectly clean. This was the nurse's doing, of course. It is part of her duty to make the bed as only a thoroughly-trained nurse can, and to wash the patient when that is necessary. It is said that in many cases the whole household catches the infection of cleanliness, and dates a great improvement in that respect from the time of nurse's visit.

The conscientious care of the nurse in these respects was shown in a marked degree in another case we visited. The mistress of the house, as well as the baby she held in her arms, was decidedly grimy, but the patient, an old woman of seventy-seven, helpless as an infant, who was slipping down towards the "strait and dreadful pass of death," was perfectly neat and clean. The nurse had just been there, and was to come again in the evening to see to her, and so make the passage through the dark valley as easy as possible.

From there we went to a house where a little girl, a large-eyed, pale creature, who had just struggled through pneumonia to succumb to phthisis, lay, limp as a plucked daisy, in her mother's arms. The matron had a toy for her, and kind, playful words to rouse her from her languor, and the promise of flannel garments and other comforts her parents—respectable people, but destitute through the present scarcity of employment—could not afford to give her.

Another serious case was that of a man who had suffered from typhoid fever, and now seemed threatened with some brain disease. His wife, a pretty woman still, though her hair was grey, trim, and neat, and brave-spirited, bade us welcome, though we interrupted her at the dinner she had paused in her washing to eat. It was a scant dinner, only potatoes, but what of that? Some one had brought her a piece of fowl and some beef-tea for "him"—the weak, thin husband, sadly depressed about his own condition, to tend whom, even with the nurse's help, must have made heavy demands on her courage and strength. But it was something to her to have him there, at home—not taken away to a hospital.

The last case we visited was an old woman, rendered helpless through two ulcerated legs, who dwelt in the garret of an old house in Stepney Square. A very bare garret it was, with few pretensions to comfort, and a very feeble occupant, though the wounds were getting slowly better under the nurse's care. But the old woman had one pet, one treasure. Not a cat! No, animal pets are not for the very poor; they have no food to spare for such. The possession of a cat, or still more of a licensed dog, implies a certain amount of prosperity. The old woman's treasure was a little fern, growing and thriving greenly in its drear surroundings, and she told us how its size had increased since she had it, and how it cheered her to see it.

And as I write these scanty notes of a morning's walk through one parish of one district watched over by the East London Nursing Society, it seems to me that I might compare it to this fern, growing and increasing in a sad and barren place, and in its growth bringing comfort and cheer.

